# Nondestructive Monitoring of Textile-Reinforced Cementitious Composites Subjected to Freeze–Thaw Cycles

**DOI:** 10.3390/ma17246232

**Published:** 2024-12-20

**Authors:** Nicolas Ospitia, Ali Pourkazemi, Eleni Tsangouri, Thaer Tayeh, Johan H. Stiens, Dimitrios G. Aggelis

**Affiliations:** 1 Department of Mechanics of Materials and Constructions, Faculty of Engineering, Vrije Universiteit Brussel, B-1050 Brussels, Belgium; eleni.tsangouri@vub.be (E.T.); thaer.tayeh@vub.be (T.T.); dimitrios.aggelis@vub.be (D.G.A.); 2 Magnel-Vandepitte Laboratory, Department of Structural Engineering and Building Materials, Faculty of Engineering and Architecture, Ghent University, B-9052 Gent, Belgium; 3 Department of Electronics and Informatics ETRO-IR, Faculty of Engineering, Vrije Universiteit Brussel, B-1050 Brussels, Belgium; apourkaz@etrovub.be (A.P.); jstiens@etrovub.be (J.H.S.); 4 Laboratoire de Mécanique des Matériaux du Génie Civil (L2MGC), Cergy Paris Université, 5 Mail Gay-Lussac, 95000 Neuville-sur-Oise, France

**Keywords:** TRC, UPV, MMW spectrometry, freeze–thaw, NDT

## Abstract

Cementitious materials are susceptible to damage not only from mechanical loading, but also from environmental (physical, chemical, and biological) factors. For Textile-Reinforced Cementitious (TRC) composites, durability poses a significant challenge, and a reliable method to assess long-term performance is still lacking. Among various durability attacks, freeze–thaw can induce internal cracking within the cementitious matrix, and weaken the textile–matrix bond. Such cracks result from hydraulic, osmotic, and crystallization pressure arising from the thermal cycles, leading to a reduction in the stiffness in the TRC composites. Early detection of freeze–thaw deterioration can significantly reduce the cost of repair, which is only possible through periodic, full-field monitoring of the composite. Full-field monitoring provides a comprehensive view of the damage distribution, offering valuable insights into the causes and progression of damage. The crack location, size, and pattern give more information than that offered by single-point measurement. While visual inspections are commonly employed for crack assessment, they are often time-consuming. Technological advances now enable crack pattern classification based on high-quality surface images; however, these methods only provide information limited to the surface. Elastic wave-based non-destructive testing (NDT) methods are highly sensitive to the material’s mechanical properties, and therefore are widely used for damage monitoring. On the other hand, electromagnetic wave-based NDTs offer the advantage of fast, non-contact measurements. Micro- and millimeter wave frequencies offer a balance of high resolution and wave penetration, although they have not yet been sufficiently explored for detecting damage in cementitious composites. In this study, TRC specimens were subjected to up to 150 freeze–thaw cycles and monitored using a combination of active elastic and electromagnetic wave-based NDT mapping methods. For this purpose, transmission measurements were conducted at multiple points, with ultrasonic pulse velocity (UPV) employed as a benchmark and, for the first time, millimeter wave (MMW) spectrometry applied. This multi-modal mapping approach enabled the tracking of damage progression, and the identification of degraded zones.

## 1. Introduction

Textile-Reinforced Cementitious (TRC) composites are advanced, thin, and lightweight materials that combine a fine-grained mortar matrix with reinforced with high-strength textiles typically made of materials such as carbon, glass, or aramid. While the mechanical properties of TRC composites have been widely studied [1,2,3], the long-term durability of TRC composites remains a critical concern. These composites can also degrade due to external sources such as physical, chemical, and/or biological factors [4,5]. In the literature, durability studies have mainly focused on chemical attacks, freeze–thaw, or elevated temperatures. For chemical attacks, studies have focused on marine, alkaline, and acidic environments. Freeze–thaw has been investigated in numerous studies [6,7]; however, the lack of a standardized method for TRC composites has resulted in a high variation in results [8]. Durability remains a significant concern, and there is as yet no reliable method to evaluate the long-term behavior of TRC composites [9].

Freeze–thaw is a type of durability attack in which an increasing number of cycles generate internal stresses due to hydraulic, osmotic, and crystallization pressures that can develop into cracks, and the deterioration of the bond between the textiles and the cementitious matrix, resulting in a reduced durability. The early detection of freeze–thaw is crucial, as it can significantly reduce repair costs.

Unlike single-point measurements, full-field crack visualization provides a comprehensive view of the damage distribution, offering deeper insight into the causes and progression of damage [10]. Detailed information on the location, size, and pattern of the cracks enhances the understanding of structural degradation mechanisms, which might be overlooked with single-point measurements. This approach supports more efficient maintenance and repair, thereby expanding the structure’s lifespan.

Non-destructive testing (NDT) techniques are widely known for their ability to detect and characterize damage in cementitious media [11]. Among these, ultrasonic methods excel due to the strong correlation between the elastic wave propagation and the mechanical properties of the measured material [12]. Ultrasound has found numerous applications in the construction industry, including cement hydration monitoring [13,14], stiffness estimation [15], and damage detection [16,17,18]. In parallel, electromagnetic wave-based NDTs in micro- and millimeter wavelengths present a good compromise between a high resolution and the penetration of the incident electromagnetic (EM) wave into the material [19,20]. For this reason, these frequencies have been recently explored for a range of applications in cementitious media, including for the evaluation of the water/cement and sand/cement ratio [21,22,23], the prediction of compressive strength [24], damage monitoring in complex composite structures [25], tracking the hydration process [26,27,28], and the measurement of hardened properties [29,30], among others. However, to the authors’ knowledge, the TRC composites’ durability under freeze–thaw conditions has not yet been evaluated with elastic and/or EM NDTs.

In this study, non-destructive elastic and electromagnetic mapping techniques are employed to identify and quantify the freeze–thaw degradation of the TRC facings. Elastic wave mapping is performed using UPV, a widely established method that serves as a benchmark for MMW spectrometry results.

## 2. Materials and Methods

The TRC specimens’ (10 × 80 × 480 mm^3^) matrix compositions are shown in Table 1. Strong cement (CEM I 52.5N, from Holcim, Obourg, Belgium) was used with a clinker content higher than 95% [31]. The river sand (0/2 mm) was sieved to a maximum aperture of 0.850 mm to remove impurities. Glass textiles with a styrene–butadiene coating [32] served as reinforcement, with a volume fraction of 1.14%. The textiles are positioned at the mid-thickness of the TRC composites. The superplasticizer Sika Viscocrete-20 Gold (from Sika, Nazareth, Belgium) was used to give more workability to the cementitious matrix while maintaining water content. Understanding the effect of a superplasticizer in the freeze–thaw resistance of cementitious media is not the purpose of the study; however, previous studies have shown improved freeze–thaw resistance for samples with superplasticizer, by allowing the w/c ratio to reduce [33,34].

The mortar was prepared by first mixing the cement with the sand for 2 min at a low mixing speed. Water and superplasticizer were then added and mixed continuously for two more minutes at low speed and a final 1 min at high speed. Immediately after mixing, half of the mortar was poured into the wooden molds and vibrated for 90 s. Afterwards, the textiles were placed, and the remaining mortar was poured, and vibrated once again for 90 s. All TRC specimens were covered in plastic foil for at least 28 days for curing. Additionally, prior to any UPV and MMW measurements, the TRC samples were submerged in water at 20 °C for 24 h. The tensile properties of the textile reinforcement SITgrid200 are included in Table 2.

### 2.1. Freeze–Thaw

**Figure 1:**
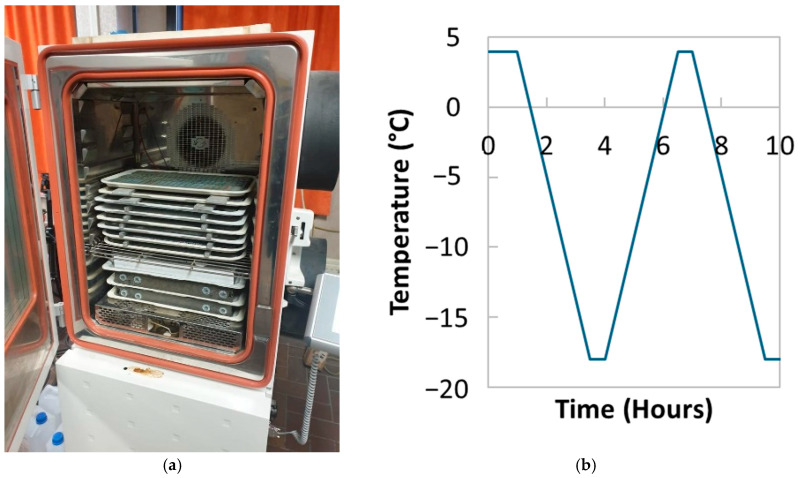
(**a**) Experimental setup and (**b**) freeze–thaw cycles [30].

The TRC specimens were exposed to rapid freeze–thaw cycles in an air-cooled Votsch VC 4018 climate chamber following the ASTM recommendations [35]. For this purpose, the TRC specimens were fully immersed in water during freezing and thawing in plastic trays, as shown in Figure 1a. Prior to the first cycle, the samples were conditioned at 4 °C for 1 h. Each cycle consisted of 2.5 h at 4 to −18 °C, 30 min at a constant temperature of −18 °C, 2.5 h at −18 to 4 °C, and finally 30 min at 4 °C (as depicted in Figure 1b). The specimens underwent a total of 150 cycles, with measurements taken every 50 cycles. In total, 18 TRC specimens were used, 12 tested in four-point bending, and 6 for UPV and MMW spectrometry.

### 2.2. Ultrasonic Pulse Velocity (UPV)

**Figure 2:**
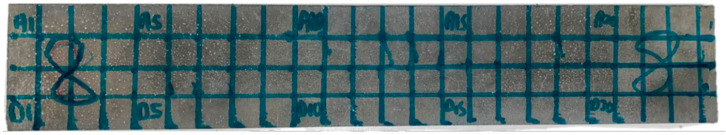
TRC composite measurement point distribution.

To assess the distribution of UPV through the thickness of the TRC specimens, a grid of 92 points—arranged in 4 rows and 23 columns—was marked on each specimen, as shown in Figure 2. The experimental setup included two resonant piezoelectric sensors at 150 kHz (emitter–receiver configuration). The emitter was connected to an Agilent 20 MHz waveform function generator, set to emit a single 150 kHz sinusoidal pulse with an amplitude of 10 V. No coupling agent was used in this case, as it would be absorbed in the external pores of the cementitious matrix, obstructing the water entry, and thus interfering with the freeze–thaw deterioration, in addition to impacting the MMW measurements.

### 2.3. MMW Spectrometry

**Figure 3:**
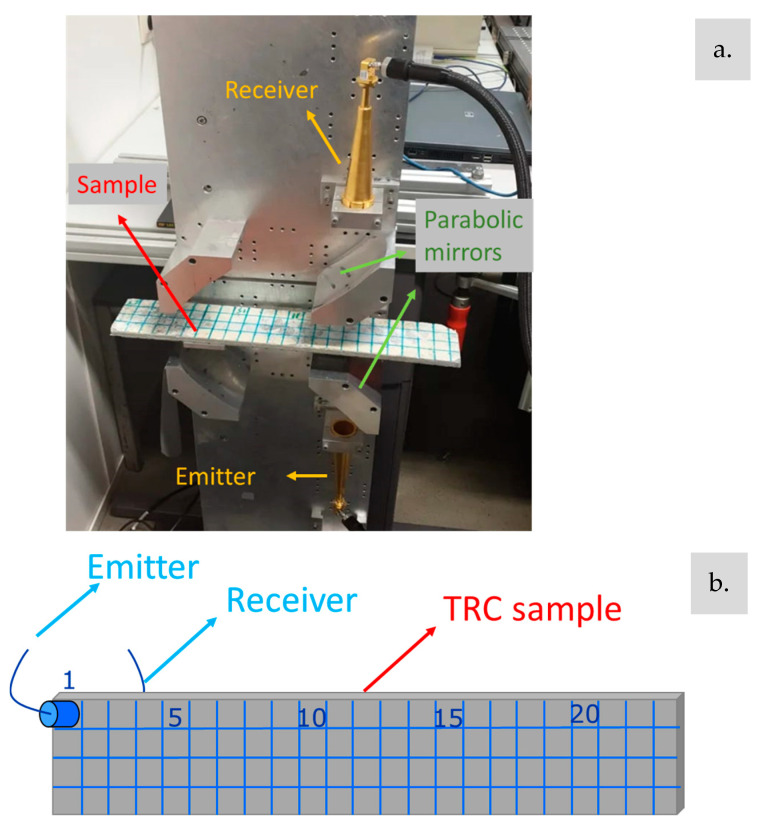
Experimental setup for (**a**) MMW spectrometry and (**b**) UPV.

The MMW experimental setup (see Figure 3a) consisted of a Keysight PNA Network Analyzer N557B (from Keysight, Santa Rosa, CA, USA), operating within a selected frequency range of 46–66 GHz, divided into 1601 frequency points. Corrugated horn antennas were used to transmit electromagnetic waves from a waveguide into free space, generating a Gaussian beam that was directed into a quasi-optical bench with four-focal parabolic mirrors. Following calibration, the PNA accurately measured both reflection and transmission at a stable room temperature of 18 ± 2 °C.

For permittivity calculations, it was assumed that the specimen’s thickness remained constant after the freeze–thaw process induced damage. As outlined in [26], a single-layer algorithm, based on wave propagation paths, as described by [36], was applied. In this approach, the summation of propagation paths in transmission corresponds to S_21_, which by definition is the forward gain, represented as the voltage at the receiver divided over the incident voltage from the emitter. In this case, each sample had 23 measuring positions, which corresponded to the 23 columns shown in Figure 2 and Figure 3b.

### 2.4. Four-Point Bending

**Figure 4:**
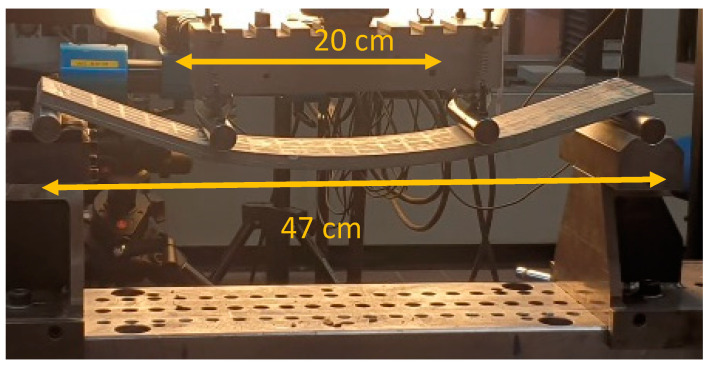
Four-point bending of TRC specimens.

Every 50 freeze–thaw cycles, the mass of each TRC specimen was recorded, followed by monitoring using MMW spectrometry and UPV. After these measurements, three specimens were removed from the climate chamber and subjected to four-point bending tests. This procedure was repeated for up to 150 cycles. The TRC specimens were tested under displacement-controlled quasi-static four-point bending at a rate of 2 mm/min (see Figure 4) using an INSTRON 5885H machine (From Instron, Ile-de France, France). In total, 12 specimens were tested in bending.

## 3. Results and Discussion

### 3.1. Freeze–Thaw

**Figure 5:**
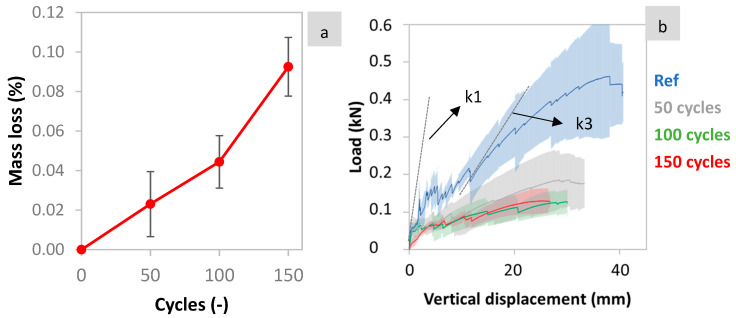

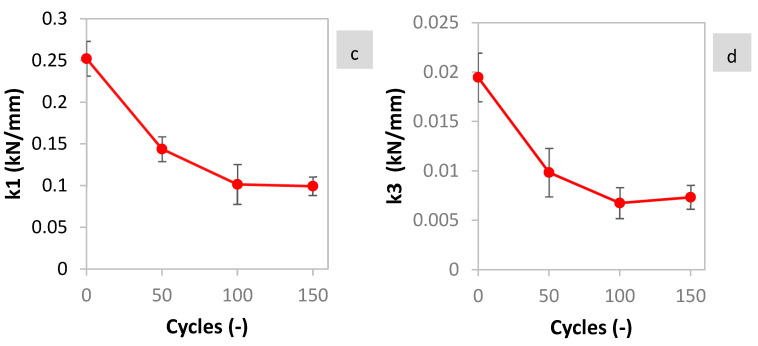
(**a**) Mass loss of TRC specimens vs. freeze–thaw cycles and (**b**) average load vs. vertical displacement (**c**) k1 and (**d**) k3 stiffness.

**Figure 6:**
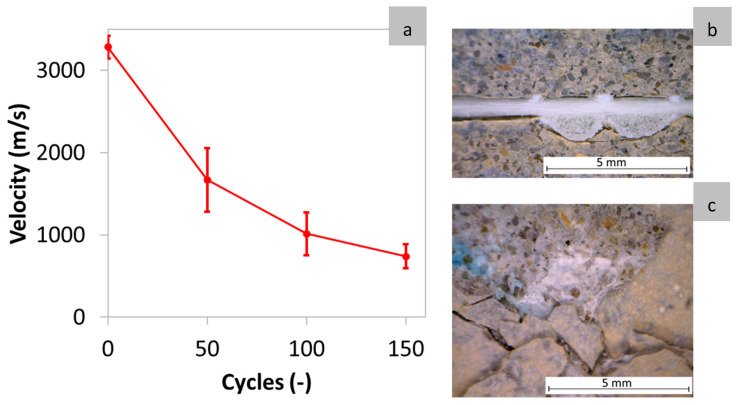
(**a**) Average UPV versus freeze–thaw cycles, (**b**) textile debonding, and (**c**) degraded surface shown as pop-outs caused by 50 freeze–thaw cycles.

Figure 5a shows the average percentage of mass loss of the TRC sandwich beams subjected to varying numbers of freeze–thaw cycles, along with the corresponding standard deviation. The results show the degradation of the cementitious matrix, as evidenced by the increasing mass loss with the number of freeze–thaw cycles. The mass loss exceeded 9% at 150 cycles. Relatively high standard deviation values are attributed to the composite nature of the TRC composites, which, as with other cementitious composites, present a high heterogeneity. Therefore, the variation of properties is always expected to be higher than in homogeneous media. Figure 5b shows the average load versus vertical displacement curves, with the corresponding standard deviation, for TRC specimens subjected to 0, 50, 100, and 150 freeze–thaw cycles based on three samples per condition. The data show that after just 50 freeze–thaw cycles, there is already a reduction of 60% of the maximum bending load of the composite. Presumably, this reduction has its roots in the degradation of the matrix (such as surface spalling, internal cracking) and its bond loss with the textiles (see Figure 6b). Similar degradation of the bond and matrix has been observed in previous studies [3]. From 50 to 100 freeze–thaw cycles, the maximum load continues to decrease, but with a smaller impact on the maximum load (around 30%, with respect to the max load at 50 cycles).

Similarly to [37], bending stiffness was quantified as the slope of the load versus vertical displacement curve during the bending test, and denoted as k (kN/mm). This slope was calculated by linear fitting for 10–60% of the load before the first crack for k1. And for k3, the slope was calculated from the start of the 3rd stage, until just before the following load drop. k1 (see Figure 5c) and k3 (see Figure 5d) represent the stiffness of the first and third stage, respectively. The k1 stiffness is dominated by the properties of the matrix, while k3 stiffness is influenced by the mechanical properties of the textile reinforcement, and the bond with the matrix. The results showed a strong decrease of 42% and 49% for both k1 and k3 during the first 50 freeze–thaw cycles, followed by smaller decreases of 28% and 25% from 50 to 100, with stiffness stabilizing after 100 cycles. These findings align with the observed reduction in the average maximum load and the overall degradation of the specimens.

The variations in UPV with increasing freeze–thaw cycles are shown in Figure 6a. The initial UPV transmission velocity measured in the reference beams was on average 3282 m/s. After 50 freeze–thaw cycles, this velocity drops by 50%, indicating the composite’s high sensitivity to freeze–thaw degradation. By 100 cycles, the velocity had dropped by 70% relative to the reference, and by 150 cycles, it had declined further to 77%. These reductions in velocity are attributed to the degradation of the cementitious matrix, which manifested as cracking parallel to the surface, delamination (see Figure 6b), and the deterioration of the TRC surface (see Figure 6c). This significant reduction in velocity is consistent with the decrease in the maximum load, k1, and k3. The reduction in k1 is likely due to matrix degradation, while the decrease in k3 reflects debonding between the matrix and the textiles. Thus, the through-transmission velocity appears to be a reliable indicator for damage caused by freeze–thaw cycles in thin TRC specimens. It is nonetheless acknowledged that the discrimination of degradation mechanisms is not possible with a single value of UPV.

**Figure 7:**
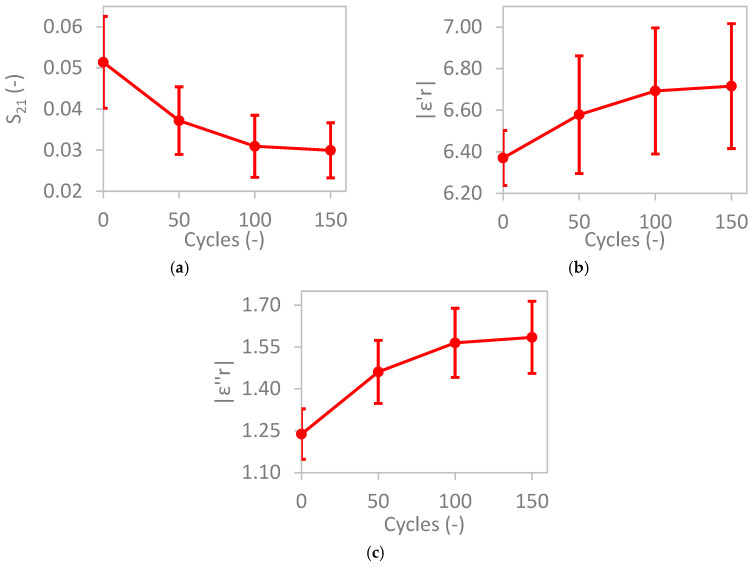
Average (**a**) S_21_, (**b**) real (ε′r), and (**c**) imaginary components of relative permittivity (ε″r) for TRC specimens subjected to freeze–thaw cycles.

Figure 7a shows the average EM transmission (S_21_, at a frequency of 55 GHz) for 10 mm thick TRC specimens as a function of freeze–thaw cycles, measured at a stable temperature of 18 ± 2 °C. The data show a consistent decrease in EM transmission with increasing freeze–thaw cycles. To further characterize material properties, the dielectric permittivity was calculated from S_21_ (Figure 7b,c). The magnitude of both the real (ε′_r_) and the imaginary (ε″_r_) components of permittivity increased with the number of freeze–thaw cycles. This behavior is attributed to the formation of cracks and voids within the cementitious matrix, which allowed greater water ingress under saturated conditions. This led to a significant increase in the composite’s effective permittivity, with a much stronger impact (4× to 5×) on the imaginary than on the real component. It is worth noting that an uneven reduction in the surface such as with spalling, or scaling, introduces a combined effect. On one side, it results in a reduction in the thickness of the specimen and, consequently, increased transmission. On the other hand, scattering produced by an uneven surface would have the opposite effect. However, under saturated conditions, the increased porosity appears to be the governing factor.

**Figure 8:**
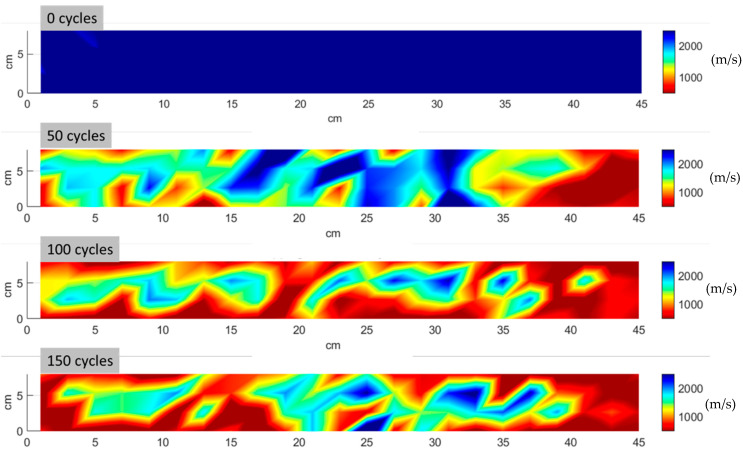
Distribution of UPV for 0, 50, 100, and 150 freeze–thaw cycles.

**Figure 9:**
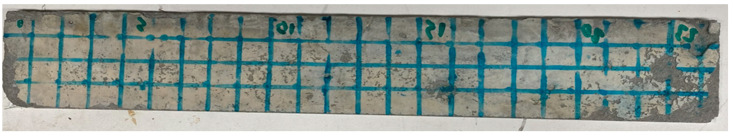
Indicative TRC specimen after 50 freeze–thaw cycles.

Figure 8 shows the velocity distribution obtained from 92 measuring points for a representative sample at intervals of 50 freeze–thaw cycles. Consistent with the average UPV results shown in Figure 6a, the data reveal a clear decline in velocity with an increasing number of freeze–thaw cycles. Moreover, the distribution of damage is not uniform, as zones with lower velocities seem to be concentrated on the sides of the TRC specimens, evidencing that damage propagates from the outer edges inwards. Figure 9 shows the photograph of the same indicative TRC specimen after 50 freeze–thaw cycles. Even visual inspection of the specimen’s surface indicates that the edges are more degraded, in agreement with the UPV map at 50 cycles.

**Figure 10:**
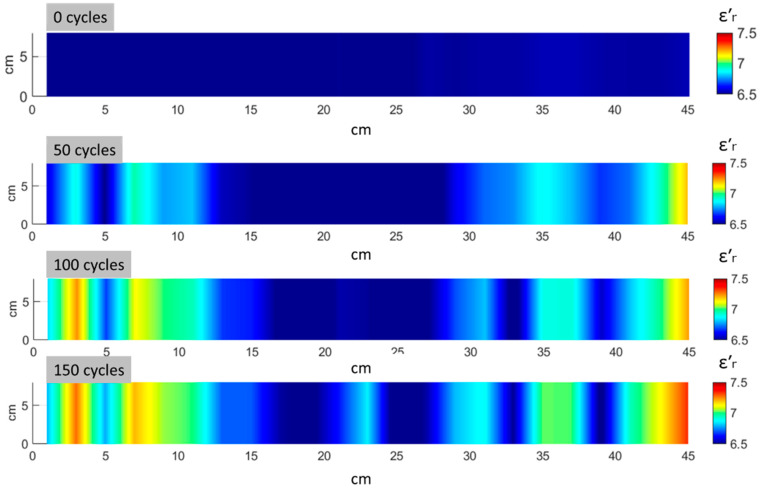
Distribution of ε′r for 0, 50, 100, and 150 freeze–thaw cycles.

**Figure 11:**
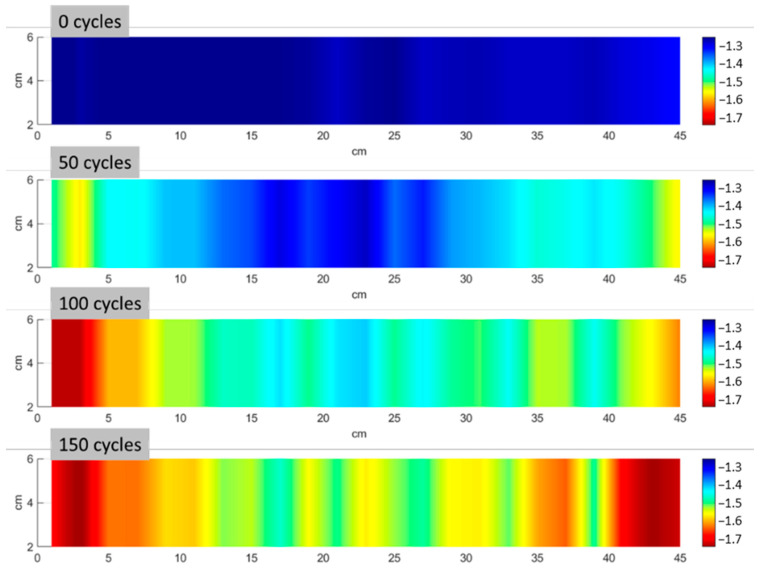
Distribution of ε″r for 0, 50, 100, and 150 freeze–thaw cycles.

Figure 10 and Figure 11 show the distribution of ε′r and ε″r components of permittivity across 23 measuring points for a representative sample at intervals of 50 freeze-thaw cycles. Areas marked in red indicate a high ε′r and ε″r, corresponding to zones with a greater degree of damage, as confirmed by visual inspection and lower UPV values. The MMW mapping reveals that areas with elevated permittivity are concentrated along the specimen edges, highlighting the directional nature of the damage. This observation aligns with the damage patterns revealed by both UPV mapping (Figure 8) and visual inspection (Figure 9). Furthermore, as the number of freeze–thaw cycles increases, the areas with a high real and imaginary permittivity expand. The agreement between the MMW results and UPV demonstrates the potential of the MMW technique for the contactless detection of freeze–thawing effects. The most pronounced results obtained in the change in the imaginary part of the permittivity.

### 3.2. Analysis on Effect of Humidity and Permittivity

**Figure 12:**
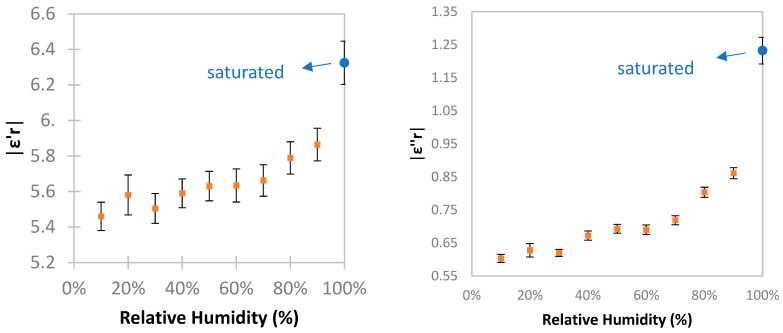
Average real and imaginary permittivity of TRC specimen with varying RH.

Humidity can dramatically affect the measured permittivity of TRC composites. For this reason, in order to provide a general understanding of this effect, and as a preliminary step towards applying MMW spectroscopy under different humidity conditions, the MMW permittivity of three reference TRC specimens (10 × 80 × 480 mm^3^) was measured at different relative humidity (RH) levels (Figure 12). The specimens were conditioned in a climate chamber at 20 °C for 24 h at each RH level starting from saturated conditions, and decreasing in 10% intervals down to 10% RH. The results, as expected, show an increase in the magnitude of both the real and imaginary permittivity of the composite with increasing RH, attributable to the high permittivity of water. The standard deviation of the calculated permittivity ranged between 2 and 3%. The point in blue in Figure 12 represents the TRC specimen in fully water-saturated conditions. From an RH of 10% up to saturated conditions, ε′r increases about 15%, while ε″r increases about 100%. The influence of humidity in the MMW measurements highlights the need for its consideration. This is crucial to ensure the accuracy and broader applicability of the technique.

## 4. Conclusions

Non-destructive mapping was conducted to detect damage and characterize its distribution in TRC specimens. This approach enabled the identification of the affected areas, the assessment of damage severity, and the localization of the zones requiring repair.

A combination of active elastic and electromagnetic wave-based mapping NDT methods was applied. Both methods consisted of performing through-transmission measurements in a series of points, obtaining a distribution of UPV as benchmark, and for the first time, permittivity maps to evaluate the freeze–thaw degradation in TRC composites. This multimodal approach enabled precise damage localization and revealed the direction of the degradation. The main conclusion points are summarized below:

- The complex permittivity proved to be a useful parameter to quantify freeze–thaw degradation. Under saturated conditions, the permittivity increased due to internal cracking and surface degradation.
- UPV demonstrated a high sensitivity to freeze–thaw cycles, with velocity decreasing by approximately 60% after the first 50 cycles. UPV results aligned with MMW findings, which showed more pronounced damage-related changes near the specimen edges.
- Both permittivity and UPV exhibited greater sensitivity to early-stage freeze–thaw damage compared to mass loss, which was used to quantify the damage produced by freeze–thaw cycles.
- Humidity significantly influences the permittivity of cementitious media. Higher relative humidity correlates with increased permittivity. This effect was quantified for reference TRC specimens as a first step to increase the applicability of the technique. However, further research is still required.

## Figures and Tables

**Table 1:** Mortar mix composition by weight.

Cement type I 52.5 N	1
River sand	2
Water	0.45
Superplasticizer	0.5%

**Table 2:** Tensile properties of SITgrid200.

	SIT Grid 200	Standard Deviation
**σ_max_ (MPa)**	979.10	22.04
**E-modulus (GPa)**	69.57	0.34

## Data Availability

The data presented in this study are available on request from the corresponding author due to privacy/ethical.
